# Seroprevalence of Brucellosis in Butchers, Veterinarians and Slaughterhouse Workers in Hamadan, Western Iran

**Published:** 2018-02-10

**Authors:** Mojgan Mamani, Mohammad Mahdi Majzoobi, Fariba Keramat, Nesa Varmaghani, Abbas Moghimbeigi

**Affiliations:** ^1^Department of Infectious Diseases, School of Medicine, Hamadan University of Medical Sciences, Hamadan, Iran; ^2^Brucellosis Research Center, Hamadan University of Medical Sciences, Hamadan, Islamic Republic of Iran; ^3^Modeling of Noncommunicable Research Center, Department of Biostatistics, School of Public Health, Hamadan University of Medical Sciences, Hamadan, Islamic Republic of Iran

**Keywords:** Slaughter houses, Veterinarians, Prevalence, Brucellosis, Iran

## Abstract

**Background:** Brucellosis is a zoonotic disease caused by Brucella species via infected domestic animals. In endemic areas, certain occupations such as veterinarians, butchers, and slaughterhouse workers are considered high risk regarding brucellosis. We evaluated the seroprevalence of brucellosis in high-risk occupations in Hamadan, West of Iran.

**Study design:** a cross-sectional study.

**Methods:** Overall, 218 participants from 2014 to 2015 were enrolled. A questionnaire including demographic data, length of employment, and using personal protective equipment was completed for each of them. Then, blood samples were taken and sent to Hamadan Health Center to be tested by Wright or standard tube agglutination (STA). In addition, samples with positive Wright test were examined by 2- mercaptoethanol (2ME) test. Then, seropositive participants were evaluated for clinical manifestations of brucellosis. All collected data were analyzed by SPSS ver. 16.

**Results:** The mean age of the participants was 42.79±11.16 yr and all seropositive cases were male. Based on Wright ≥1/80 and 2ME ≥1/40, seroprevalence of brucellosis was 13.3% and 12.3%, respectively. The use of personal protective equipment was low among individuals with or without brucellosis. Myalgia, fatigue, back pain, joint pain, night sweats, fever, malaise, and headache were common symptoms in seropositive cases. Moreover, 20.6% of the seropositive participants were asymptomatic.

**Conclusions:** Prevalence of brucellosis in these occupational groups and symptomatic disease in significant numbers of them was high, so periodic clinical examinations in these groups seems to be essential for brucellosis surveillance system.

## Introduction

Brucellosis, an important zoonotic disease in developing countries, is directly or indirectly transmitted from infected animals to human. Due to its diverse clinical manifestations, diagnosis of brucellosis usually needs to be confirmed by bacteriological or serological tests^1^.

Although food hygiene has greatly improved, brucellosis still exists in many parts of the world especially in developing countries^2,3^. The disease is endemic and a major health concern in the Middle East (including Iran), India, Mexico, Central, and South America^4^. Brucellosis is seen in all provinces of Iran, but the highest incidence rate of the disease has been reported in Azerbaijan, Hamadan, Lorestan, Markazi, and Kermanshah provinces^5^. In industrialized countries, brucellosis is an occupational disease mainly prevalent among middle-aged men exposed to infected milk and livestock products^6^.

Owing to its different manifestations, serological tests are the only positive findings in most cases. Common serological tests for the diagnosis of brucellosis in Iran include Rose Bengal, rapid tube agglutination, standard tube agglutination (STA), Coombs-Wright, 2-mercaptoethanol (2-ME), and ELISA for both immunoglobulin M (IgM) and immunoglobulin (IgG) antibodies^7^.

Various studies have reported a higher prevalence of brucellosis in people working with animal and their products. In a study, the prevalence of brucellosis among abattoir based on Rose Bengal plate and complement tests reported 4.7% and 1.3%, respectively^8^. High IgG titer serology in abattoir workers indicates brucellosis infection in them and highlights the necessity of not only preventive health measures in slaughterhouses but also elimination of infected animals and mass vaccination of healthy ones ^9^. Given the high prevalence of acute and chronic brucellosis among butchers and veterinary staff as well as general population and transmission of brucellosis through contact with infected livestock and dairy products, further research is required to clarify the prevalence of brucellosis and its seropositivity in high-risk groups, in different parts of Iran.

Identifying an appropriate screening tool in high-risk occupational groups, such as butchers and slaughterhouse workers, based on serological tests can be effective in reducing the transmission and spread of disease. Therefore, we evaluated the seroprevalence of brucellosis in butchers, veterinarian and slaughterhouse workers in Hamadan, West of Iran.

## Methods

This cross-sectional study was conducted to evaluate the seroprevalence of brucellosis in high-risk individuals including butchers, slaughterhouse workers, and veterinarians in Hamadan, West of Iran 2014 to 2015. The only inclusion criterion was having a high-risk job for brucellosis. Based on the formula for estimating sample size and assuming the prevalence of brucellosis as 0.098 based on previous study ^10^, 95% confidence interval, and an absolute error of 0.05, the minimum numbers of butchers, slaughterhouse workers, and veterinarians were calculated as 81, 47 and 15, respectively, but given the high tendency of the volunteers, the number of participants reach 218. Moreover, the butcher shop is rarely found in the villages and also logistic restrictions, sampling was limited to Hamadan district.

After holding a briefing session in coordination with the Butchers’ Guild and Veterinary Office, all butchers were invited to the union butcher place and after explanation regarding brucellosis infection by researcher, informed consent was obtained from participants and blood samples were taken from them. By the same way, blood samples were also taken from slaughterhouse workers who work in central slaughterhouse in Hamadan City. Moreover, voluntary veterinarians gave blood samples in person in the laboratory, too.

A designed questionnaire including demographic characteristics, use of personal protective equipment, consumption of unpasteurized dairy products, history of brucellosis, family history of brucellosis, place of residence (urban, rural), and long-time contact with animals was completed for each participant and then 10 ml blood sample was taken and sent to the laboratory of Hamadan Health Center. All blood samples were tested with STA test, but Coombs-Wright and 2ME tests were done on samples with negative and positive STA results, respectively. The all used kits tests were prepared by Pasteur Institute of Iran. Seropositive participants were referred to the Infectious Diseases Clinic for clinical examination by physician. The data was recorded in a relevant checklist.

Based on national guideline, Wright≥ 1/80^5^ and international value^1^, Wright≥1/160, serological analysis of samples was performed twice.

### Ethical consideration

The study protocol was approved by Ethics Committee of Hamadan University of Medical Sciences with code of IR.UMSHA.REC.1394.229.

### Statistical analysis

All data were analyzed using SPSS 16.0 (SPSS Inc., Chicago, IL, USA). Quantitative data were presented as mean and standard deviation (SD). Qualitative data were reported as frequency and percentage. Independent *t* -tests were used to compare normalized quantitative data. Chi-square tests were applied to analyze the qualitative data. *P* -value less than 0.05 was considered statistically significant in all tests.

## Results

The present study was conducted on 218 participants including 112 butchers, 86 slaughterhouse workers and 20 veterinarians. Most participants (n = 213) were male and only five were female. The mean age of the participants was 42.79±11.16 yr (range: 18-75 yr). All seropositive cases were male. Based on the national and international reference values, 29 (13.3%) and 10 (4.6%) subjects were positive, respectively. Of 29 participants who had positive Wright test, 27 (93.1%) were also positive for 2ME test. Ninety percent of seropositive cases had Wright ≥1/160 and 2ME ≥1/80.

Among 29 seropositive subjects for brucellosis, 24 cases (82.8%) lived in urban areas and 5(2.17%) lived in rural areas. More than 80% of participants had junior high school or lower education. Seroprevalence of brucellosis among butchers, veterinarians, and slaughterhouse workers was 17%, 8.1%, and 15%, respectively. Moreover, 18 (62.1%) of the seropositive subjects had a history of consuming rural dairy products and 16 (55.5%) had a history of brucellosis (Table 1).

**Table 1:** Demographic characteristics in butchers, slaughterhouse workers, and veterinarians based on serology of Brucella

**Variables**	**Wright, n (%)**			**Wright, n (%)**		
	**<1/80**	**≥1/80**	***P*** **v alue**	**<1/160**	**≥1/160**	***P*** **value**
Gender			0.376			0.620
Male	184 (97.0)	29 (100)		203 (97.0)	10 (100)	
Female	5 (3.0)	-		5 (3.0)	-	
Occupation			0.188			0.423
Butchers	93 (83.0)	19 (17.0)		105 (50.0)	7 (6.2)	
Slaughterhouse workers	79 (91.9)	7 (8.1)		84 (40.0)	2 (2.3)	
Veterinarians	17 (85.0)	3 (15.0)		19 (10.0)	1 (5.0)	
Place of residence			0.295			0.386
Urban	169 (89.0)	24 (82.8)		185 (89.0)	8 (80.0)	
Rural	20 (11.0)	5 (17.2)		23 (11.0)	2 (20.0)	
Education			0.412			0.804
Illiterate	13 (7.0)	2 (6.9)		14 (6.7)	1 (10.0)	
Elementary school	73 (38.6)	10 (34.5)		80 (38.4)	3 (30.0)	
Junior high school	50 (26.5)	12 (41.5)		59 (28.0)	3 (30.0)	
High school diploma	34 (7.0)	2 (6.9)		35 (16.8)	1 (10.0)	
Academic degree	19 (20.9)	3 (10.3)		20 (10.1)	2 (20.0)	
Using unpasteurized dairy products			0.249			0.525
Yes	137 (72.0)	18 (62.9)		147 (70.0)	8 (80.0)	
No	52 (28.0)	11 (37.1)		61 (30.0)	2 (20.0)	
History of brucellosis			0.001			0.366
Yes	44 (23.0)	16 (55.2)		56 (27.0)	4 (40.0)	
No	145 (77.0)	13 (44.8)		152 (73.0)	6 (60.0)	

The use of personal protective equipment such as masks and goggles was generally low in these occupational groups. In fact, only one of them used all the five pieces of personal protective equipment. Of 112 studied butchers, 16, 5, 38, 48, and 72 individuals used masks, goggles, gloves, boots, and apron, respectively. Furthermore, 10, 4, 20, 75, and 53 slaughterhouse workers used the above-mentioned pieces of equipment, respectively. The corresponding values among the veterinarians were 12, 7, 12, 6, and 9, respectively. Additionally, of 189 individuals with negative STA results, only two (1.1%) had positive Coombs-Wright test (Table 2).

**Table 2:** Seroprevalence of brucellosis in butchers, slaughterhouse workers, and veterinarians based on the use of personal protective equipment

**Personal protective equipment**	**Wright ≥1/80, n (%)**			**Wright ≥1/160, n (%)**		
	**Butchers**	**Slaughterhouse workers**	**Veterinarians**	**Butchers**	**Slaughterhouse workers**	**Veterinarians**
Mask						
Yes	1 (2.5)	1 (14.2)	2 (66.5)	0 (0.0)	0 (0.0)	1 (100)
No	18 (94.8)	6 (85.7)	1 (33.5)	7 (100)	2 (100)	0 (0.0)
Goggles						
Yes	1 (2.5)	0 (0.0)	2 (66.5)	0 (0.0)	0 (0.0)	1 (100)
No	18 (94.8)	7 (100)	1 (33.5)	7 (100)	2 (100)	0 (0.0)
Gloves						
Yes	5 (26.3)	1 (14.2)	1 (33.5)	0 (0.0)	0 (0.0)	1 (100)
No	14 (73.7)	6 (85.7)	2 (66.5)	7 (100)	2 (100)	0 (0.0)
Boots						
Yes	7 (36.8)	6 (85.7)	1 (33.5)	2 (28.5)	1 (50.0)	1 (100)
No	12 (63.2)	1 (14.2)	2 (66.5)	5 (71.5)	1 (50.0)	0 (0.0)
Apron						
Yes	11 (58.0)	4(57.0)	1 (33.5)	3 (43.0)	0 (0.0)	1 (100)
No	8 (42.0)	3(43.0)	2 (66.5)	4 (57.0)	2 (100)	0 (0.0)

The most common symptoms in seropositive individuals (Wright ≥1/80) were myalgia, fatigue, low back pain, arthralgia, fever, chills, night sweats and weakness. No cases of orchitis or testicle pain were observed among the male participants. Moreover, fatigue, low back pain, arthralgia, myalgia, and headache were the most common symptoms among individuals with Wright ≥1/160.

## Discussion

In this study, 29 out of 218 participants (13.3%) had seropositive results (Wright ≥1/80) which had the highest frequency in the butchers, veterinarians, and slaughterhouse workers, respectively. Only 1.1% of individuals with negative STA test had positive Coombs-Wright test. As a result, the seroprevalence of disease was 14.4%, and 6.4% of the participants had Wright ≥1/160. This rate increased to 7.5% by including individuals with positive Coombs-Wright and negative STA test. The findings of the study were much higher than that of the general population of Hamadan (90.7 per 100,000 or 0.09%)^11^.

Overall, 500000 individuals throughout the world are diagnosed with brucellosis each year. For every confirmed case of brucellosis, there are 26 undiagnosed and non-reported, brucellosis cases^12^. Therefore, the prevalence of brucellosis in the general population is about 5 per 1000. In the present study, the prevalence of brucellosis in high-risk groups was significantly higher than in general population.

In Bangladesh, the seroprevalence of brucellosis was 11.11% among veterinary personnel^13^. In Pakistan, the seroprevalence of brucellosis among high-risk individuals was 6.9%^14^. However, in a cross-sectional study, 21.7% of the evaluated individuals in Lahore, Pakistan were IgG-positive. This high percentage was due to the high sensitivity of ELISA test^15^. The seroprevalence of brucellosis was reported in slaughterhouse workers and people living in rural areas in northern Iran as 8.9% and 5.5%, respectively^10^. The seroprevalence of brucellosis was calculated in high-risk occupational groups in Kazeroon, southern Iran, as 7.8%^6^. In Shiraz, Iran, the seroprevalence of the disease in slaughters, butchers, and the general population was 20%, 4%, and 2%, respectively ^16^. The seroprevalence of brucellosis was reported in people with high-risk occupations including butchers, slaughterhouse workers in two separate studies in Kurdistan and Sistan and Baloochestan as 12% and 7.9%, respectively^17,18^. These differences in seroprevalence rates can be attributed to dissimilar prevalence of brucellosis in various areas and the sensitivity of different screening methods.

In the present study, 24 out of 29 individuals (82.8%) with positive STA test lived in urban areas and only five (17.2%) lived in rural areas. In contrast, a study on normal population in Turkey reported the seroprevalence of brucellosis in rural areas to be higher than in urban areas^19^. Moreover, in Hamadan most patients with brucellosis were from rural areas^20-22^. This inconsistency is not surprising since this study was conducted among high-risk groups in urban area in Hamadan.

In our study, the highest prevalence of brucellosis was seen in people with junior high school or lower level of education. This highlights the role of lower awareness and less attention to the effect of personal protective equipment in the prevalence of the disease. In addition, a case-control study in Arak (Iran) identified low level of education as an important risk factor for brucellosis^23^.

The present study also investigated the prevalence of brucellosis based on the use of personal protective equipment. The use of masks, gloves, goggles, and boots was clearly lower in patients with brucellosis. However, due to the generally limited use of personal protective equipment among all participants, the difference between individuals with and without the disease was not significant. In another study, the low seroprevalence of brucellosis among butchers was justified by the suitable use of personal protective equipment ^17^. Moreover, our study investigated the prevalence of brucellosis based on the use of personal protective equipment in different occupational groups. Brucellosis was more prevalent among butchers that did not use masks, gloves, goggles, and boots; the slaughterhouse workers who did not use mask, gloves, and goggles and also in the veterinarians who did not use boots, apron, and goggles. Moreover, the prevalence of brucellosis was higher in participants consuming rural unpasteurized dairy products. Several studies have identified the consumption of unpasteurized dairy products are as a risk factor for brucellosis^2,8,23,24^. Therefore, the high rates of consuming unpasteurized dairy products in the present study might have served as a confounding factor. However, the relationship between the consumption of unpasteurized dairy products and seropositivity was not significant.

The seroprevalence of brucellosis was significantly higher in individuals with a history of brucellosis. This can be attributed to these individuals’ constant contact with contaminated products. While a positive family history was introduced as a risk factor for brucellosis in previous research ^23^, only a small percentage of seropositive individuals in this study had a positive family history of brucellosis. Meanwhile, the absence of a positive family history in our study highlights the importance of occupation in brucellosis. The present study did not find a significant relationship between positive STA test and occupation in different age groups. This can indicate a lack of compliance with safety rules in all age and occupational groups.

In the current study, most individuals with positive STA test (93.1%) had positive 2ME test. This rate was lower in a previous study in Shiraz, Iran^16^. Moreover, based on our findings, 1.1% of individuals with negative STA test had positive Coombs-Wright test. A study in Uremia (Iran) detected acute and chronic brucellosis in 8.3% and 13.2% of the participants, respectively^9^. These findings can indicate higher acute form of the disease among our participants.

The most common symptoms in seropositive cases (Wright≥ 1/80) were myalgia, fatigue, low back pain, arthralgia, fever, chills, night sweats, and weakness. No cases of chitisor or testicle pain were observed in this study. The frequency of symptoms in individuals with a Wright ≥ 1/160 was also investigated and low back pain; arthralgia, myalgia, and headache were identified as most common symptoms among them. In Shiraz, Iran the most common symptoms in individuals with positive STA test were fever, arthralgia, headache, myalgia and low back pain^6^, while in another study, the most common symptoms of brucellosis were fever (77.4%) and arthralgia (70%)^25^.

In our study, six seropositive cases, including one with a titer above 1/160 and five with titerabove1/80, were asymptomatic. In other words, 20.6% of patients with a titer above 1/80 and 10% of those with a titer above 1/160 were completely asymptomatic. The only complaint in two symptomatic patients was low back pain. One case had the highest titer (Wright=1/1280) without any symptoms except fatigue. This finding can suggest the subclinical cases of brucellosis.

The limitation of this study was the low number of slaughterhouse workers and veterinarians in Hamadan city. We recommend doing a study in more population of high-risk groups for brucellosis in

## Conclusions

The prevalence of brucellosis, as an important public health problem, was high in high-risk occupational groups including veterinarians, butchers and slaughterhouse workers. Furthermore, a significant percentage of individuals were asymptomatic. Hamadan Province is an endemic area for brucellosis, screening high-risk groups for brucellosis can be helpful. In addition, periodic clinical examinations in these groups seem to be essential for brucellosis surveillance system.

## Acknowledgements

The authors would like to acknowledge butchers guild of Hamadan City, as facilitators towards the success of this research.

## Conflict of interest statement

The authors declare no competing interests.

## Funding

Funding for this research was provided by the Vince-Chancellor for Research and Technology of Hamadan University of Medical Sciences.

## Highlights

- Brucellosis is an important public health problem.
- Prevalence of brucellosis was high in high-risk occupational groups
- Screening of high-risk groups for brucellosis can be helpful.
